# ATP-P2X7R pathway activation limits the Tfh cell compartment during pediatric RSV infection

**DOI:** 10.3389/fimmu.2024.1397098

**Published:** 2024-07-09

**Authors:** Constanza Russo, Silvina Raiden, Silvia Algieri, María José Bruera, Norberto De Carli, Mariam Sarli, Héctor Cairoli, Leonardo De Lillo, Ivanna Morales, Vanesa Seery, Adrián Otero, Inés Sananez, Nancy Simaz, Gisela Alfiero, Gabriela Rubino, Néstor Moya, Luisa Aedo Portela, Mauro Herrero, Marina Blanco, Misael Salcedo Pereira, Fernando Ferrero, Jorge Geffner, Lourdes Arruvito

**Affiliations:** ^1^ Instituto de Investigaciones Biomédicas en Retrovirus y SIDA, Facultad de Medicina, Universidad de Buenos Aires- Consejo Nacional de Investigaciones Científicas y Técnicas, Buenos Aires, Argentina; ^2^ Departamento de Medicina, Hospital General de Niños Pedro de Elizalde, Buenos Aires, Argentina; ^3^ Servicio de Pediatría, Hospital Nacional Profesor Alejandro Posadas, Buenos Aires, Argentina; ^4^ Servicio de Pediatría, Clínica del Niño de Quilmes, Buenos Aires, Argentina

**Keywords:** children, RSV, ATP, Tfh cell, antibodies

## Abstract

**Background:**

Follicular helper T cells (Tfh) are pivotal in B cell responses. Activation of the purinergic receptor P2X7 on Tfh cells regulates their activity. We investigated the ATP-P2X7R axis in circulating Tfh (cTfh) cells during Respiratory Syncytial Virus (RSV) infection.

**Methods:**

We analyzed two cohorts: children with RSV infection (moderate, n=30; severe, n=21) and healthy children (n=23). We utilized ELISA to quantify the levels of PreF RSV protein-specific IgG antibodies, IL-21 cytokine, and soluble P2X7R (sP2X7R) in both plasma and nasopharyngeal aspirates (NPA). Additionally, luminometry was employed to determine ATP levels in plasma, NPA and supernatant culture. The frequency of cTfh cells, P2X7R expression, and plasmablasts were assessed by flow cytometry. To evaluate apoptosis, proliferation, and IL-21 production by cTfh cells, we cultured PBMCs in the presence of Bz-ATP and/or P2X7R antagonist (KN-62) and a flow cytometry analysis was performed.

**Results:**

In children with severe RSV disease, we observed diminished titers of neutralizing anti-PreF IgG antibodies. Additionally, severe infections, compared to moderate cases, were associated with fewer cTfh cells and reduced plasma levels of IL-21. Our investigation revealed dysregulation in the ATP-P2X7R pathway during RSV infection. This was characterized by elevated ATP levels in both plasma and NPA samples, increased expression of P2X7R on cTfh cells, lower levels of sP2X7R, and heightened ATP release from PBMCs upon stimulation, particularly evident in severe cases. Importantly, ATP exposure decreased cTfh proliferative response and IL-21 production, while promoting their apoptosis. The P2X7R antagonist KN-62 mitigated these effects. Furthermore, disease severity positively correlated with ATP levels in plasma and NPA samples and inversely correlated with cTfh frequency.

**Conclusion:**

Our findings indicate that activation of the ATP-P2X7R pathway during RSV infection may contribute to limiting the cTfh cell compartment by promoting cell death and dysfunction, ultimately leading to increased disease severity.

## 1 Introduction

Respiratory syncytial virus (RSV) stands as the leading cause of childhood hospitalization attributed to bronchiolitis, accounting for 3 million hospitalizations and over 100,000 deaths annually in children under 5 years (1). Most fatalities occur in low- and middle-income countries, where children lack proper access to healthcare (2). Effective protection against severe RSV infection can be achieved through the passive transfer of neutralizing IgG antibodies targeting the RSV fusion (F) protein (3–5). After decades of research (6), the first maternal vaccine against RSV has recently gained approval, demonstrating its capability to prevent serious illness in infants from birth to 6 months of age (7, 8).

Follicular helper T cells (Tfh) are essential for adaptive immune response against viral infection and vaccination, helping B cells to generate high-affinity antibodies and to differentiate into memory B cells (9, 10). Both of these responses are strongly dependent on the production of IL-21 by Tfh cells. While Tfh cells were initially identified in tonsils, they can also be found in peripheral blood (circulating Tfh, cTfh cells). Similar to tissue Tfh cells, cTfh cells are capable of providing helper signals to B cells (11–13). Recent reports have indicated that changes in the frequency, phenotype and function of cTfh cells are associated with the quality of the antibody response in infectious diseases (14). For instance, the frequency of PD-1+CXCR5+CD4+ cTfh cells has shown to correlate with the production of broadly neutralizing antibodies in people living with HIV (15–17) while the disfunction of the cTfh compartment identifies poor responders to influenza vaccine (18). Notably, a significant expansion and activation of cTfh were documented during the critical phase of Dengue fever (19). Our recent findings also indicate that a low frequency of cTfh in children with severe COVID-19 is associated with a poor antibody response (20). Although neutralizing antibodies play a crucial role in controlling RSV disease, the function of the Tfh cell compartment during this infection has been relatively underexplored.

Extracellular ATP is virtually absent in the interstitium of tissues under physiological conditions but accumulates at sites of tissue injury (21). Its effects are primarily mediated by plasma membrane purinergic receptors (22). *P2X7R*, which encodes the ATP-gated P2X7 receptor, stands out as a signature gene of effector T cell subsets and is highly expressed on Tfh cells (23). Stimulation of P2X7R promotes Th1/Th17 polarization of CD4+ T cells, conversion of Tregs into Th17 cells and dysfunction of the Tfh cell compartment (24–29). We hypothesize that the inflammatory response induced by RSV infection might lead to the release of extracellular nucleotides from stressed, damaged, or dying cells, enabling the purinergic system to modulate the T cell compartment. In this study, we investigated the potential role of the ATP-P2X7R pathway in the modulation of the cTfh cell compartment during pediatric RSV infection.

## 2 Materials and methods

### 2.1 Study subjects

The study included 74 children under 24 months of age who were admitted to the Hospital General de Niños Pedro de Elizalde, Hospital Nacional Prof. Alejandro Posadas, and Clínica del Niño de Quilmes during the 2022–2023 respiratory seasons. Two main cohorts were established: 1) children with confirmed RSV determined by direct immunofluorescence of nasopharyngeal aspirates (NPAs, n=51), and 2) healthy children (n=23) admitted for minor scheduled surgery with no airway infections in the preceding 4 weeks. Disease severity was categorized using the clinical disease severity score (CDSS) as mild (0–5), moderate (6–8), or severe (9–12) based on the modified Tal score at the time of sampling. The CDSS for all admitted patients was ≥ 7. No children with mild disease were included. Patient characteristics are detailed in **Table 1**.

**Table 1:** Characteristics of study cohorts.

	RSV children		Healthy children	
	Moderate	Severe		
	N=30	N=21	N=23	
Demographic characteristics				
Age, month, median (range)	5 (1–23)	5 (1–21)	7 (4–20)	
<6, n (%)	14 (47)	14 (67)	12 (52)	
>6, n (%)	16 (53)	7 (33)	11 (48)	
Female sex, n (%)	12 (40)	9 (43)	16 (70)	
Days from symptom onset to admission, median (range)	4 (1–6)	4 (1–6)	NA	
Days from symptom onset to sampling, median (range)	6 (2–10)	6 (2–10)	NA	
Severity				
CDSS, range ^a^	7–8	9–12	NA	
Comorbidities				
None, n (%)	28 (93)	16 (76)	21 (91)	
Renal disorder^b^, n (%)	0	1 (5)	0	
Prematurity, n (%)	2 (7)	2 (9)	2 (9)	
Genetic disorder^c^, n (%)	0	1 (5)	0	
Cardiac disorder^d^, n (%)	0	1 (5)	0	
Coinfections				
None, n (%)	28 (93)	15 (71)	23 (100)	
Viral, type, n (%)	1 (3)	4 (19)	NA	
Adenovirus, n	0	3		
Rhinovirus, n	1	1		
Bacterial, type, n (%)	1 (3)	2 (10)	NA	
Staphylococcus, n	1	1		
Pseudomonas, n	0	1		
Clinical status				
Pneumonia, n (%)	5 (17)	7 (33)	NA	
PICU admission, n (%)	0	15 (71)	NA	**** Mod vs Sev
Oxygen requirement, n (%)	6 (20)	13 (62)	NA	** Mod vs Sev
Mechanical ventilation, n (%)	0	11 (52)	NA	**** Mod vs Sev
Laboratory				
WBC, counts/mm3, mean ± SD	9479 ± 3709	8909 ± 3523	10900 ± 3856	
Lymphocytes, %, mean ± SD	41.1 ± 14	38.9 ± 16	37.9 ± 10	
CD4, %, mean ± SD	40.7 ± 14.9	37.3 ± 13.1	40.1 ± 12.4	
CD19, %, mean ± SD	29.6 ± 9.6	30.4 ± 10.4	23.5 ± 12.9	

CDSS, clinical disease severity score; NA, not applicable; PICU, pediatric intensive care unit; RSV, respiratory syncytial virus; WBC, white blood cells. ^a^CDSS was calculated using the modified Tal score (0–5 mild, 6–8 moderate, and 9–12 severe); ^b^ renal insufficiency; ^c^Down syndrome; ^d^Congenital heart disease. Fisher’s exact test, Chi-Square test or Kruskall-Wallis test followed by Dunn’s multiple comparison test were used. Only significant p values are shown. **p<0.01 ****p<0.0001.

### 2.2 Sample processing

Blood samples (0.5–1 mL) were collected into EDTA tubes within 1–4 days of hospital admission. After centrifugation at 1000 rpm for 10 minutes, the plasma fraction was separated and stored at -80°C until needed, while the remaining blood sample was immediately utilized for cell isolation. When clinically permissible, NPAs were collected from RSV children and processed within 3 hours.

### 2.3 Isolation of peripheral blood mononuclear cells

Peripheral blood mononuclear cells (PBMCs) were obtained from blood samples by Ficoll-Paque gradient centrifugation (Cytiva). Cells were washed, and suspended in culture medium (RPMI 1640, Sigma-Aldrich) supplemented with 10% heat-inactivated fetal calf serum (FCS, Sigma-Aldrich), 2 mM L-Glutamine (Sigma-Aldrich), and penicillin-streptomycin (Sigma-Aldrich).

### 2.4 Cells and virus

HEp-2 cells (ATCC® CCL-23™) were cultured in DMEM (GIBCO) containing 10% FCS, 2mM L-Glutamine, and penicillin-streptomycin. Experiments utilized the human RSV subtype A, Long strain, propagated in HEp-2 cells with DMEM supplemented with 2% FCS. For virus isolation, infected cell monolayers were scraped, briefly vortexed, pelleted, and resuspended in fresh medium. RSV was purified through ultracentrifugation on a 35% sucrose layer at 4 °C, followed by resuspension in DMEM 10% trehalose (Sigma-Aldrich). The purified virus was stored at -80°C until use.

### 2.5 ELISA

To quantify plasma levels of PreF RSV protein-specific IgG antibodies, we conducted an indirect ELISA following established procedures (4). Nickel-coated 96-well plates (Pierce) were overnight coated at 4°C with 2.5 μg/mL PreF protein (SC-TM, generously provided by Dr. Mark E. Peeples). After blocking, plasma samples were diluted 1:3,000 in blocking buffer and incubated for 2 hours at RT. Following washing steps, plates were incubated for 1 hour at RT with biotinylated anti-human IgG (1:20,000, Jackson Immunoresearch) and streptavidin-HRP for 30 minutes at RT. Then, TMB Substrate (BD Biosciences) was applied, and absorbance was measured at 450 nm. Samples were normalized to a calibration curve of intravenous immunoglobulin (IVIg, 50 mg/mL, Laboratorio de Hemoderivados, UNC) and expressed as arbitrary units. Plasma levels of P2X7R soluble form (sP2X7R, Cusabio) and IL-21 (Biolegend) were determined following the respective manufacturer’s instructions.

### 2.6 Neutralization assay

Neutralization assays were performed as previously described (30, 31). Briefly, plasma samples were heat-inactivated (56°C for 30 minutes) and subjected to serial dilutions (1/2 to 1/1024). These dilutions were then incubated at 37°C for 90 minutes in the presence of purified RSV (MOI=0.02) in DMEM with 2% FCS. The resulting mixtures were deposited onto 96-well plates, and 100 μl of 2x10^5^ HEp-2 cells were added. After 4 days, cells were fixed with 4% paraformaldehyde (Sigma-Aldrich) at 4°C for 20 minutes and stained with crystal violet solution in methanol (Sigma-Aldrich). Absorbance at 585 nm was measured using a SpectraMax i3 plate reader (Molecular Devices) with 80 reads per well. The IC50 was calculated.

### 2.7 ATP measurement

ATP levels in plasma, NPA, and supernatant culture were quantified using the CellTiter-Glo reagent (Promega) following the manufacturer’s instructions by luminometry (SpectraMax i3X, Molecular Devices) (32, 33).

### 2.8 Real-time quantitative RT-PCR

RNA from NPAs was extracted using the Chemagic Viral DNA/RNA kit following the manufacturer’s instructions (PerkinElmer). RT-qPCR was conducted using the SARS-CoV-2 PLUS ELITe MGB® Kit (ELITechGroup) on a CFX96 BioRad system. Detection of RSV (A and B) RNA was performed with human RNase P serving as an endogenous internal control. Samples were deemed positive when cycle threshold values (Ct) were below 38.

### 2.9 Cell culture

Freshly isolated PBMCs were used in all the experiments performed. To assess ATP release by cells, PBMCs at a concentration of 5x10^6^/mL were stimulated with anti-CD2/CD3/CD28 coated beads (0.3 μg/mL, Miltenyi Biotec) for 5 minutes, and the supernatant was collected. For apoptosis testing, PBMCs at 1x10^6^/mL were cultured for 24 hours in the absence or presence of 2’(3’)-O-(4-benzoylbenzoyl) ATP (300 µM, BzATP, P2X7R agonist, Sigma-Aldrich) and/or KN-62 (1 µM, P2X7R antagonist, Sigma-Aldrich). For exploring the proliferative response, 1x10^6^/mL PBMCs stimulated with anti-CD2/CD3/CD28 coated beads (0.75 μg/mL) were cultured for 3 days, with or without BzATP (100 μM) and/or KN-62 (1 µM). For quantifying IL-21 production, 1x10^6^/mL PBMCs were treated or not with BzATP (100 μM) and/or KN-62 (1 µM) for 18 hours. Subsequently, cells were stimulated with 50 ng/mL PMA and 1 μg/mL ionomycin (Sigma-Aldrich) in the presence of monensin (Biolegend) for 5 hours. Doses for BzATP and KN-62 were selected based on titration curves (28, 32). All experiments were analyzed by flow cytometry.

### 2.10 Flow cytometry

PBMCs (1x10^6^ cells) were stained at RT for 20 minutes and subsequently washed with PBS containing 1% BSA. The following monoclonal antibodies were used: anti-CD4 (Brilliant Violet 711, BD Biosciences), anti-CD45RA (FITC, Biolegend), anti-CXCR5 (Brilliant Violet 421, Biolegend), anti-PD-1 (Brilliant Violet 510, Biolegend), anti-ICOS (APC, Biolegend), anti-CD19 (APC-Cy7, Biolegend), anti-CD38 (PerCP, Biolegend), anti-CD27 (PE, BD Biosciences), anti-P2X7R (Alexa Fluor 647, Santa Cruz) and AnnexinV (FITC, Biolegend). For IL-21 detection, cells were fixed and permeabilized using Cytofix/Cytoperm and Perm/Wash buffer (BD Biosciences), followed by staining with an anti-IL-21 antibody labeled with PE (BD Biosciences). For Ki-67 detection, a similar procedure was followed using an anti-Ki-67 antibody labeled with FITC (BD Biosciences) and the BD Pharmingen™ Human FOXP3 Buffer set (BD Biosciences) for fixation and permeabilization. Data were acquired using a Northern Lights (Cytek) flow cytometer and analyzed with FlowJo 10.6.2.

### 2.11 Statistical analysis

Clinical characteristics were summarized using descriptive statistics. Categorical variables are reported as numbers and percentages. Quantitative variables are reported as medians and interquartile ranges and presented as medians and minimum to maximum in the figures. The normality of experimental data was evaluated by the Shapiro-Wilk test. Two groups were compared using the Wilcoxon signed-rank test or Mann-Whitney U test. Three or more groups were compared using the Friedman test or Kruskall-Wallis test followed by Dunn’s multiple comparison test (the method used is stated in the figure legends). Proportions were compared using the Fisher’s exact test and Chi-Square test. Correlation between two continuous variables was calculated using a Spearman correlation test. To try a multivariate approach, we conducted a logistic regression model with the severity of disease as dependent variable and including age, gender, cTfh frequency, and plasma levels of ATP and sP2X7R as independent variables. The regression model was implemented in the R environment (R Core Team, 2021). Statistical significances are indicated in the figures by asterisks as follows *p<0.05, **p<0.01, ***p<0.001 or ****p<0.0001. Analysis and visualizations were performed using GraphPad Prism.v.8 (GraphPad Software) and SPSS software v.19.0 (SPSS Corp).

## 3 Results

### 3.1 Plasma levels of IgG antibodies directed to RSV are lower in children with severe disease

**Figure 1:**
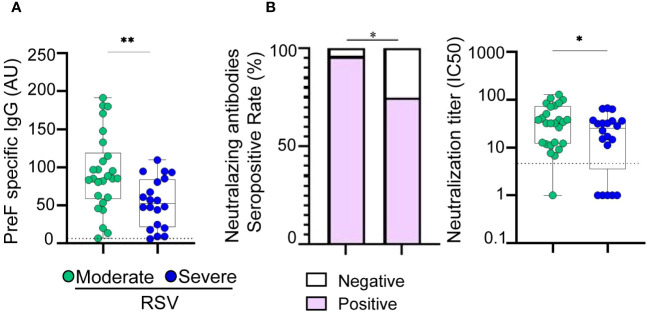
Antibody response in children with moderate and severe RSV infection. **(A)** Plasma levels of IgG antibodies directed to PreF protein of RSV of moderate (n=26) and severe (n=20) RSV infected infants quantified by ELISA. **(B)** Left: Bar graphs showing the percentage of positive samples for neutralizing activity against RSV (moderate, n=26 and severe, n=20). Right: Neutralization antibody titers against RSV determined by the reciprocal IC50 in plasma from moderate and severe RSV infants. Dotted line indicates the limit of detection value. Median and min to max of n donors are shown in **A, B** (right). P values were determined by Fisher’s exact test and Mann-Whitney U test. *p<0.05, ** p<0.01. UA., arbitrary units. Moderate (green circle), severe (blue circle). Negative (white square), positive (purple square).

The levels of IgG antibodies targeting RSV were examined in hospitalized children due to moderate and severe RSV infection aged 1 to 23 months. No children with mild disease were included. Blood samples were collected within 1–4 days after admission. Our analysis revealed that the titers of IgG antibodies directed to the PreF protein of RSV were significantly lower in children with severe disease compared to those with moderate disease (p<0.01, see **Figure 1A**). Additionally, we investigated the plasma neutralizing activity against RSV. It was observed that 96% (n=25) of children with moderate disease and 75% (n=15) of children with severe disease were positive for the presence of plasma neutralizing antibodies (p<0.05; **Figure 1B**, left). Severe cases not only exhibited a decreased rate of seropositivity but also showed lower titers of neutralizing antibodies (p<0.05, **Figure 1B**, right).

### 3.2 Children with severe RSV infection exhibit a diminished frequency of circulating follicular helper T cells and plasmablasts along with lower plasma levels of IL-21

**Figure 2:**
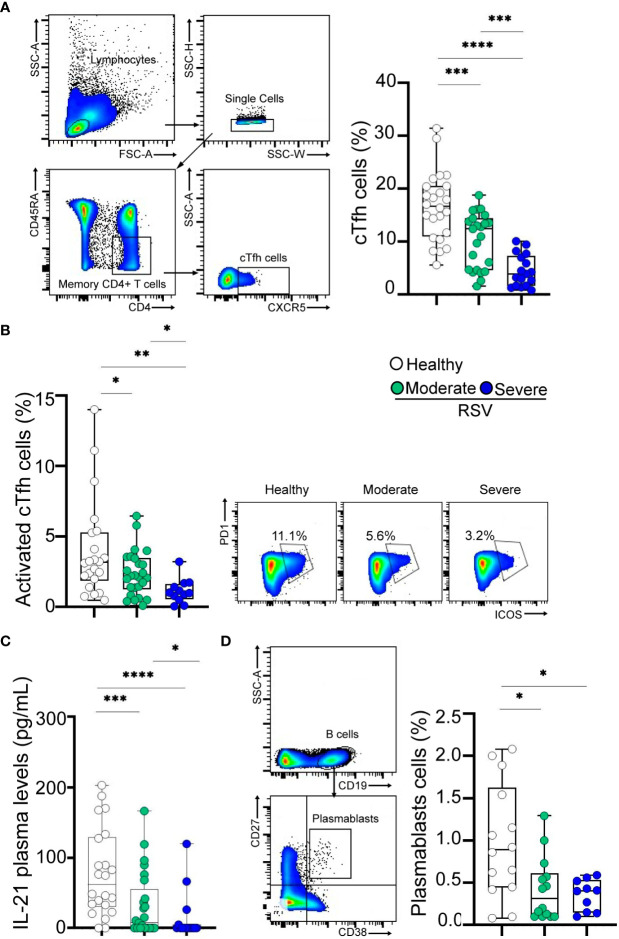
Frequency of cTfh cells and plasmablasts in RSV and healthy children. **(A)** Left: Gating strategy of cTfh cells defined as CD4+CD45RA-CXCR5+ cells. Right: Frequency of cTfh cells in healthy children (n=24) and RSV children (moderate, n=25 and severe, n=17) analyzed by flow cytometry. **(B)** Left: Frequency of activated cTfh cells defined as CD4+CD45RA-CXCR5+PD-1+ICOS+ cells in healthy children (n=21) and RSV children (moderate, n=25 and severe, n=11) analyzed by flow cytometry. Right: Representative dot plots of a donor from each cohort is shown. **(C)** Plasma levels of IL-21 in healthy children (n=22) and RSV children (moderate, n=25 and severe, n=15) quantified by ELISA. **(D)** Left: Gating strategy for plasmablasts is shown. Right: Frequency of plasmablasts defined as CD19+CD27^hi^CD38^hi^ cells in healthy children (n=14) and RSV children (moderate, n=14 and severe, n=10) analyzed by flow cytometry. Median and min to max of n donors are shown in **A** (right), **B** (left), **C, D** (right). P values were determined by Kruskall-Wallis test followed by Dunn’s multiple comparison test and Mann-Whitney U test. *p<0.05, ** p<0.01, *** p<0.001, ****p<0.0001. Moderate (green circle), severe (blue circle), healthy (white circle).

To investigate potential defects in the Tfh cell compartment associated with RSV infection, we initially examined the frequency of CXCR5+ cTfh cells (CD4+CD45RA-CXCR5+ T cells), as a correlate of lymph node Tfh cells (11). The gating strategy is shown in **Figure 2A**, left. The frequency of CD4+ T cells was comparable among children with moderate or severe conditions and healthy donors (**Table 1**, **Supplementary Figure 1**). Our results revealed a decreased frequency of cTfh cells in RSV-infected children, with those with severe disease displaying the lowest percentages (p<0.001 and p<0.0001 for moderate and severe disease, respectively, vs healthy; p<0.001 for moderate vs severe disease; **Figure 2A**, right). Similar findings were observed when assessing the frequency of activated cTfh cells, defined as CD4+CD45RA-CXCR5+PD-1+ICOS+ cells (p<0.05 and p<0.01 for moderate and severe disease, respectively, vs healthy; p<0.05 for moderate vs severe disease; **Figure 2B**, left). Representative dot plots are presented in **Figure 2B** (right). Additionally, a consistent trend was noted in the analysis of plasma IL-21 levels (p<0.001 and p<0.0001 for moderate and severe disease, respectively, compared to healthy; p<0.05 for moderate vs severe disease; **Figure 2C**). We then analyzed the circulating B cell compartment. There were no statistical differences in the frequency of B cells among children with moderate or severe RSV and healthy donors (**Table 1**, **Supplementary Figure 1**). Lastly, we examined the frequency of plasmablasts, characterized by CD19+CD27^hi^CD38^hi^ phenotype, as illustrated in **Figure 2D** (left). While plasmablast frequency was diminished in RSV-infected children compared to healthy counterparts, no significant differences were observed between children with severe and moderate infections (p<0.05; **Figure 2D**, right).

### 3.3 Children with severe disease show increased levels of extracellular ATP and P2X7R expression on cTfh

Previous studies have indicated that Tfh cells express heightened levels of P2X7R on the plasma membrane, rendering them susceptible to cell death upon exposure to ATP (23, 34).

**Figure 3:**
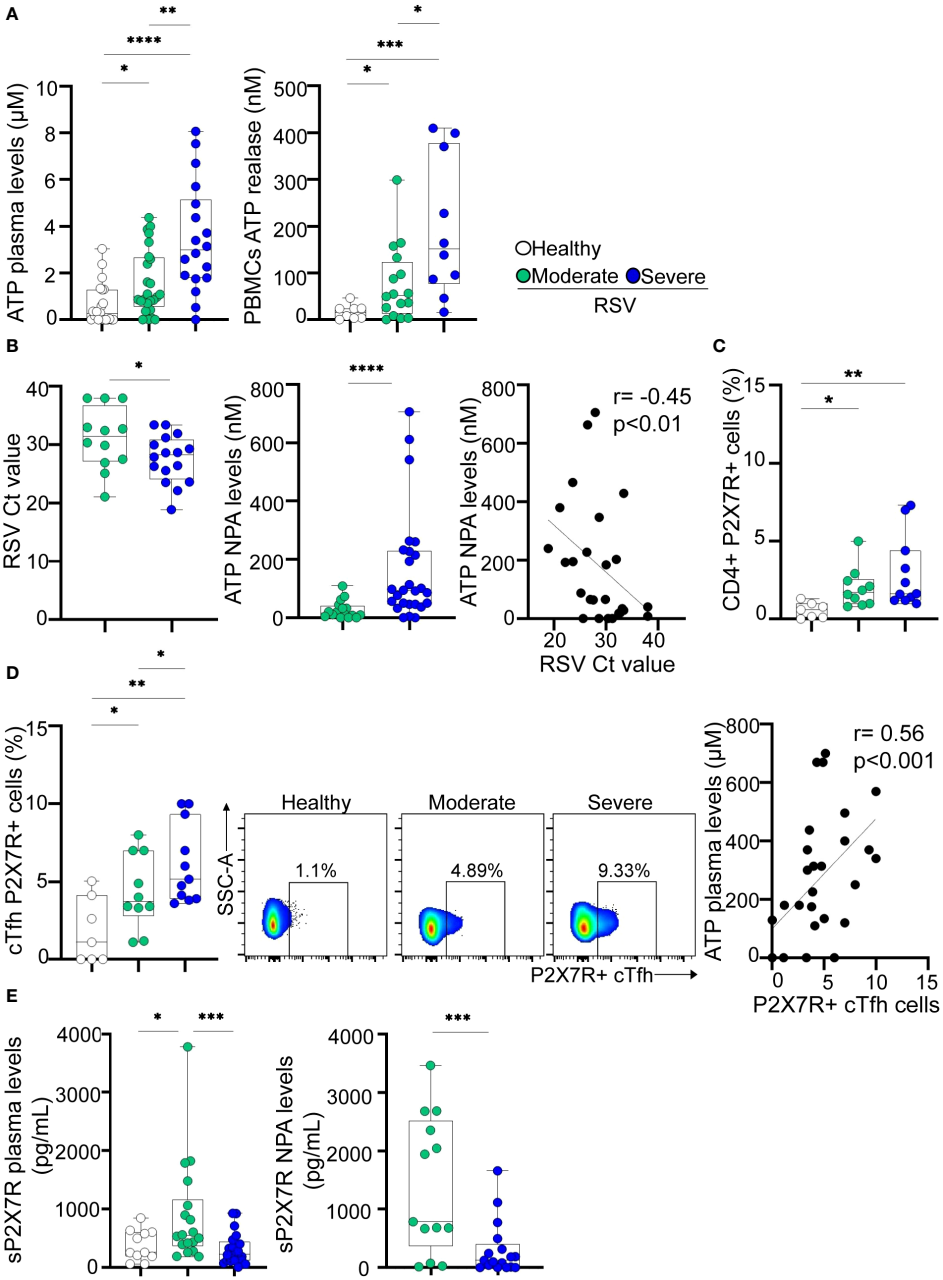
Extracellular ATP levels and P2X7R expression in RSV and healthy children. **(A)** Left: Levels of ATP in plasma from healthy children (n=21) and RSV children (moderate, n=25 and severe, n=18) measured by luminometry. Right: PBMCs (5x10^6^/mL) from healthy children (n=8), and RSV children (moderate, n=16 and severe, n=10) were stimulated with anti-CD2/CD3/CD28 coated beads (0.3 μg/mL) for 5 minutes. Levels of extracellular ATP were measured in the supernatant by luminometry. **(B)** Left: RSV Ct value distribution in NPA from moderate (n=12) and severe (n=16) RSV children quantified by RT-qPCR. Middle: Levels of ATP in NPA from moderate (n=16) and severe (n=26) RSV children measured by luminometry. Right: Graph showing the correlation between levels of ATP in NPA and RSV Ct values of RSV children (n=26). **(C)** Frequency of CD4+ T cells expressing P2X7R in healthy children (n=7) and RSV children (moderate, n=10 and severe, n=11) analyzed by flow cytometry. **(D)** Left: Frequency of cTfh expressing P2X7R in healthy children (n=7) and RSV children (moderate, n=10 and severe, n=11) analyzed by flow cytometry. Middle: Representative dot plots of P2X7R expression in a donor of each cohort is shown. Right: Graph showing the correlation between levels of ATP in plasma and the frequency of cTfh P2X7R+ cells of RSV and healthy children (n=28). **(E)** Levels of P2X7R soluble form in plasma (left; healthy children, n=11, moderate, n=18 and severe, n=21) and NPA (right; moderate, n=13 and severe, n=17) quantified by ELISA. Median and min to max of n donors are shown in **A, B** (left and middle), **C, D** (left) and **E**. P values were determined by Kruskall-Wallis test followed by Dunn’s multiple comparison test, Mann-Whitney U test and Spearman correlation test. *p<0.05, ** p<0.01, *** p<0.001, ****p<0.0001. Non-severe (green circle), severe (blue circle), healthy (white circle).

Consequently, we investigated whether the alterations observed in cTfh cells, as detailed above, were associated with increased extracellular ATP levels. Our analysis revealed elevated plasma ATP levels in RSV-infected children compared to healthy donors, with severe children exhibiting the highest values (p<0.05 and p<0.0001 for moderate and severe disease, respectively, vs healthy; p<0.01 for moderate vs severe disease; **Figure 3A**, left). Furthermore, indicative of T cell activation during RSV infection, we observed that upon stimulation, PBMCs from infected children released higher amounts of ATP compared to healthy children, with the most pronounced production observed in patients with severe disease (p<0.05 and p<0.001 for moderate and severe disease, respectively, vs healthy; p<0.05 for moderate vs severe disease; **Figure 3A**, right). Analysis of NPA samples revealed that children with severe RSV infection exhibited higher viral loads (p<0.05; **Figure 3B**, left) and elevated ATP levels (p<0.0001) compared to those with moderate infection (**Figure 3B**, middle). Notably, an inverse relationship between RSV Ct values and ATP concentrations was observed in NPA samples (**Figure 3B**, right).

The P2X7 receptor subtype is the most critical regulator of T cell development and function among the P2X family members (35). Our initial observations revealed a significant increase in P2X7R expression on CD4+ T cells from children with RSV compared to controls (p<0.05 and p<0.01 for moderate and severe disease vs healthy; **Figure 3C**). In addition, we observed higher frequencies of cTfh P2X7R+ cells in children with both moderate and severe disease compared to healthy controls (p<0.05 for moderate disease and p<0.01 for severe disease vs. healthy controls). Of note, the increase in P2X7R expression was significantly greater in children with severe RSV related to those with moderate disease (p<0.05, **Figure 3D**, left). Representative dot plots are illustrated in **Figure 3D** (middle). Moreover, a positive correlation was established between the percentage of P2X7R+ cTfh cells and plasma ATP levels (r=0.56, p<0.001; **Figure 3D**, right).

It is well known that full-length P2X7R can be released from immune cells into the circulation during inflammatory conditions, by proteolytic cleavage or associated with microvesicles derived from the membrane of different cell types (36, 37). We observed elevated levels of sP2X7R in the plasma of children with moderate disease compared to healthy donors, but such elevation was not observed in the plasma of children with severe infection (**Figure 3E**, left). Moreover, higher levels of P2X7R soluble form were detected in NPA samples from children with moderate disease compared to those with severe disease (p<0.001; **Figure 3E**, right).

### 3.4 P2X7R stimulation modulates the cTfh cell compartment in RSV infected children

**Figure 4:**
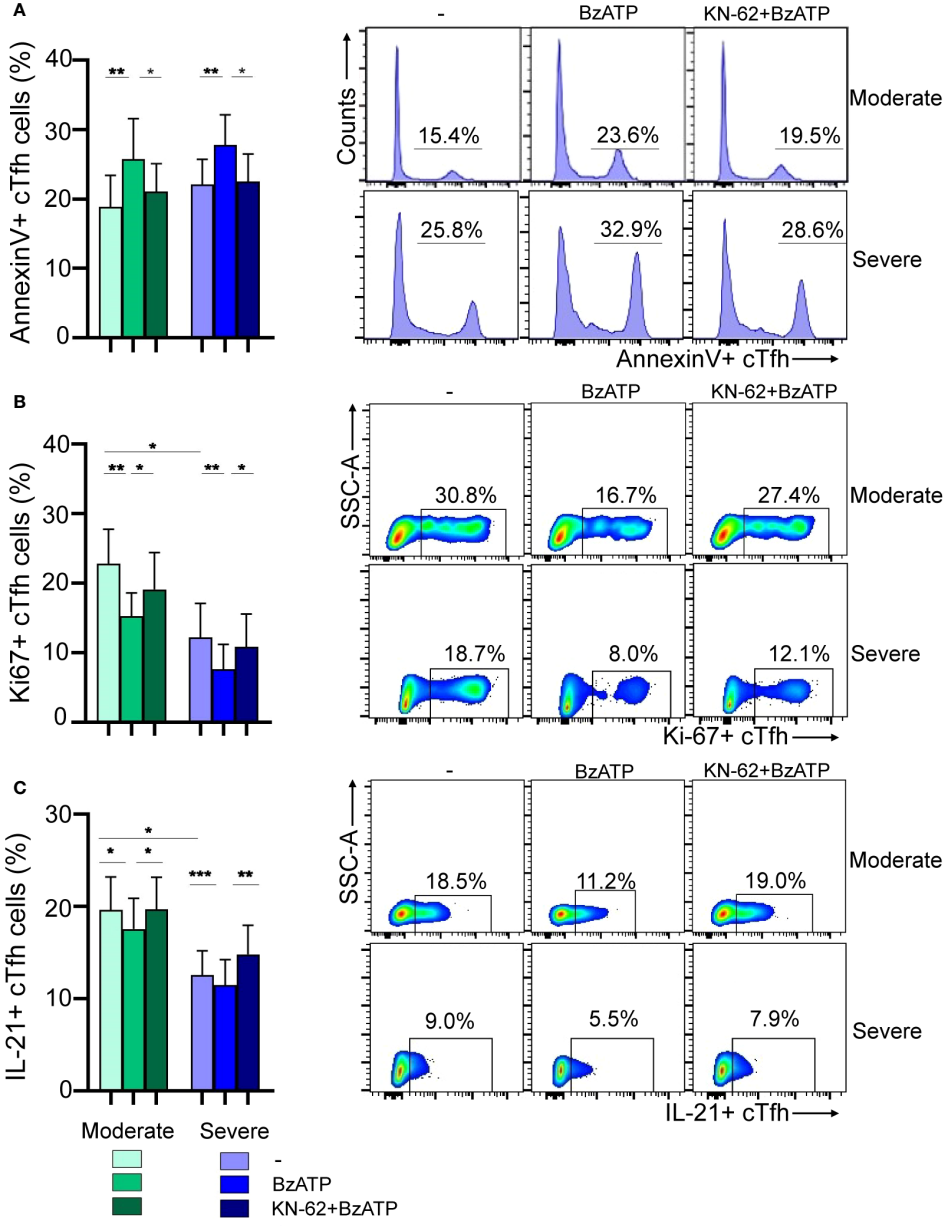
Modulation of viability and function of cTfh cells via P2X7R stimulation during RSV infection. **(A)** Left: PBMCs (1x10^6^/mL) from RSV moderate (n=9) and severe children (n=8) were incubated with BzATP (300 µM), BzATP plus KN-62 (1µM) or nontreated for 24 hours. Percentage of apoptosis of cTfh cells was analyzed by flow cytometry. Right: Representative histograms showing Annexin V+ cTfh cells in a donor of each cohort are depicted. **(B)** Left: PBMCs (1x10^6^/mL) from RSV moderate (n=10) and severe children (n=8) were stimulated with anti-CD2/CD3/CD28 coated beads (0.75 μg/mL) and treated or not with BzATP (100 μM) and/or KN-62 (1µM) and cells were culture for 3 days. Frequency of cTfh Ki-67+ cells was evaluated by flow cytometry. Right: Representative dot plots showing Ki-67+ cTfh cells in a donor of each cohort are shown. **(C)** Left: PBMCs from RSV moderate (n=9) and severe children (n=10) were treated or not with BzATP (100 μM) and/or KN-62 (1µM) for 24 hours. Afterward, were re-stimulated with PMA and Ionomycin in the presence of monensin for 5 hours. Percentage of cTfh IL21+ cells were analyzed by flow cytometry. Right: Representative dot plots showing IL-21+ cTfh cells in a donor of each cohort are depicted. Mean ± SEM of n donors are shown in **A** (left), **B** (left) and **C** (left). P values were determined by Wilcoxon, Friedman test followed by Dunn’s multiple comparison test and Mann-Whitney U test. *p<0.05, ** p<0.01, *** p<0.001. Moderate (green squares), severe (blue squares).

Considering both the fact that P2X7R stimulation by ATP in Tfh cells promotes cell death and our own results indicating increased expression of P2X7R in cTfh cells from children with severe infection, we investigated whether Tfh cells from these children exhibited heightened susceptibility to cell death upon P2X7R stimulation. For this purpose, PBMCs obtained from children with moderate or severe disease were exposed or not, for 24 hours, to BzATP (300 µM), a potent P2X7R agonist, in the absence or presence of the P2X7R antagonist KN-62 (28, 32). Following treatment, the cells were stained with AnnexinV. We observed a similar increase in apoptosis of cTfh cells in both groups of children upon exposure to BzATP, an effect prevented by the addition of KN-62 (p<0.01 and p<0.05 for BzATP vs untreated and BzATP vs BzATP plus KN-62, respectively; **Figure 4A**, left). Representative histograms are shown in **Figure 4A** (right). Consistent with these observations, we found that the proliferative response of this cell subset induced by TCR-stimulation was substantially lower in the presence of BzATP (p<0.01), in both moderate and severe disease, and that the addition of KN-62 partially rescued the proliferative response (p<0.05; **Figure 4B**, left). Remarkably, the proliferative response of cTfh cells upon TCR-stimulation was reduced in children with severe disease respect to children with moderate disease (p<0.05). Representative dot plots are shown in **Figure 4B** (right). Finally, we investigated whether the production of IL-21, primarily mediated by cTfh cells, was affected by BzATP. A significant inhibition was observed (p<0.05 and p<0.001 for moderate and severe disease, respectively), partially rescued by KN-62 (p<0.05 and p<0.01 for moderate and severe disease, respectively; **Figure 4C**, left). Importantly, we observed a more significant decreased of IL-21 in children with severe disease compared to those with moderate symptoms (p<0.05). Representative dot plots are shown in **Figure 4C** (right).

### 3.5 Frequency of cTfh cells, plasma levels of sP2X7R, and ATP are related to disease severity

**Figure 5:**
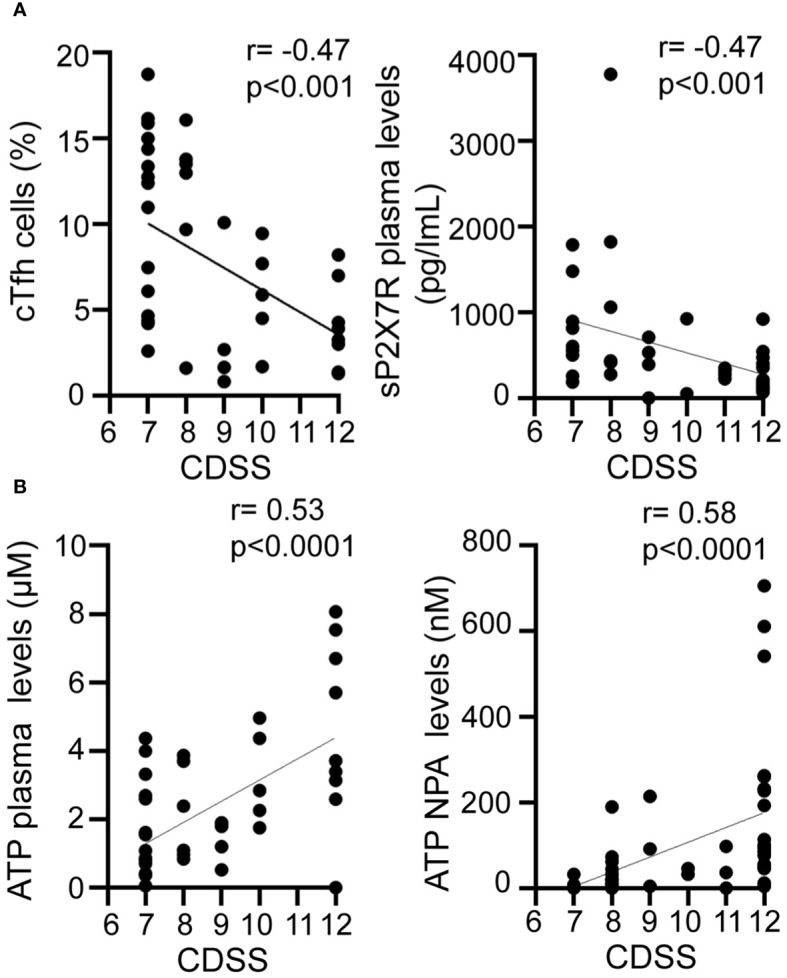
Relationship between disease severity and cTfh and purinergic signaling components. Graphs showing correlations between CDSS and frequency of cTfh cells (**A** left), P2X7R soluble form plasma level (**A** right), plasma ATP level (**B** left), NPA ATP level (**B** right) of RSV infected children (n=42). Spearman correlation test. CDSS, clinical disease severity score.

Significant gaps persist in our understanding of biomarkers that define severe disease and predict clinical outcomes during RSV infection. Our findings reveal a negative correlation of CDSS with both cTfh cell frequency (r= -0.47, p<0.001; **Figure 5A**, left) and sP2X7R plasma levels (r= -0.47, p<0.001; **Figure 5A**, right). Conversely, CDSS positively correlates with ATP levels in both plasma (r=0.53, p<0.0001; **Figure 5B**, left) and NPA (r=0.58, p<0.0001; **Figure 5B**, right). To compare disease severity as outcome, we performed a logistic regression analysis adjusting for age, gender, cTfh frequency, and plasma levels of sP2X7R and ATP as independent variables. This logistic regression model (n=42) suggested that severity of disease is associated with cTfh cell frequency (B=-0.294, p<0.05) and plasma ATP levels (B=0.877, p<0.01, **Supplementary Table 1**).

## 4 Discussion

In this study, we present findings indicating that acute RSV infection in children is linked to a decline in the frequency of cTfh. Moreover, changes in the activation state of cTfh during RSV infection are demonstrated by a reduced percentage of PD-1+ICOS+CXCR5+ cTfh in infected children compared to healthy donors. Interestingly, both cTfh and activated cTfh cell frequencies reach their lowest levels in children with severe infection, contrasting with those with moderate infection. Consistent with these observations, plasma levels of IL-21 were found to be diminished in infected children compared to healthy donors, with the lowest levels detected in children with severe infection. Tfh cells provide help to B cells, supporting the formation of germinal centers and promoting the production of high affinity IgG antibodies and memory B cells (11). While typically found in secondary lymphoid organs, circulating Tfh (cTfh) cells can also be detected in human blood. Sequencing of their TCR transcripts reveals significant clonal overlap with tonsillar Tfh cell subsets, providing valuable insights into lymphoid tissue responses (13). The frequency and phenotype of cTfh cell often undergo alterations under viral infections. For instance, a subset of circulating memory PD-1+CXCR5+CD4+ T cells has been found to correlate with the development of broadly neutralizing antibodies against HIV in a large cohort of individuals living with HIV (15). Moreover, this subpopulation has been implicated in persistent HIV-1 transcription in treated aviremic individuals (16). Children undergoing the acute phase of measles infection also exhibited an abnormal expansion of cTfh cells, which did not correlate with neutralizing IgG antibody levels (38). During the critical phase of Dengue Fever, a significant activation of cTfh cells and a positive correlation with plasmablasts have been noted (19). Finally, our recent findings indicate a poor and delayed antibody response associated with a low frequency of cTfh in children with severe COVID-19 (20).

Despite the pivotal role that play neutralizing antibodies in controlling RSV infection, there has been limited exploration of the Tfh cell compartment’s function during this infection. Remarkably, a recent study using a murine RSV infection model reported Tfh dysfunction characterized by reduced IL-21 production and diminished IL-21 receptor expression. The authors also demonstrated that blocking PD-L1 expressed by dendritic cells resulted in enhanced IL-21 production by Tfh cells, reduced lung RSV load and disease severity, and an overall improvement in the anti-RSV humoral response (39). Our findings align with these results and suggest a compromise in the Tfh compartment during human RSV infection.

The dysfunction in the Tfh compartment does not seem to be a distinctive feature exclusive to RSV infection. Indeed, serum IL-21 levels, as well as the frequency of IL-21+ cTfh cells, were lower in patients infected with HCV compared to healthy individuals. Intriguingly, it has been shown that low frequencies of IL-21+ cTfh are associated with an exhausted phenotype in CD8+ T cells during chronic HCV infection (40). Furthermore, examination of postmortem thoracic lymph nodes and spleen from individuals with acute SARS-CoV-2 infection revealed a reduced frequency of Bcl-6+ Tfh cells and the absence of germinal centers (41). Additionally, a recent study has shown that the delayed development of virus-specific Tfh cells correlates with disease severity in COVID-19 patients (42). Similarly, a dysfunctional antigen-specific Tfh cell compartment with an altered IL-21/IL-2 axis has been observed in individuals with impaired influenza vaccine responses (18).

Extracellular nucleotides and purinergic receptors play crucial roles in various cellular processes during viral infections, serving multiple functions. They can exert potent antiviral effects by enhancing interferon signaling. For instance, extracellular ADP, by activating the P2Y13 receptor, has been demonstrated to restrict the replication of various viruses, including vesicular stomatitis virus, herpes simplex virus 1, and murine leukemia virus (43). Furthermore, studies have shown the involvement of the P2X7 receptor in controlling Dengue virus infection (44, 45). However, in certain scenarios, their activity can contribute to hyperinflammatory responses and disease severity, leading to adverse outcomes, as observed in influenza (46) and COVID-19 (33, 47). Lastly, viruses such as HIV-1 use the purinergic system to favor their infection and persistence within host cells (48).

Given that bronchiolitis induced by RSV infection is associated with the sloughing and death of airway epithelial cells, along with the induction of an acute inflammatory reaction promoting tissue injury (49–51), we speculate that these processes might lead to the release of extracellular nucleotides by stressed, damaged, or dying cells. This release could enable the purinergic system to modulate the immune response during RSV infection. In a murine model of RSV infection, it was demonstrated that the infection of bronchoalveolar epithelial cells induces the release of UTP. This UTP, acting through P2Y purinergic receptors, leads to the development of bronchiolitis and pneumonia (52). In this study, we provide the first demonstration that plasma levels of ATP are elevated in children with RSV compared to healthy children. Furthermore, we observed higher levels of ATP in both plasma and NPA samples from patients with severe infection compared to those with moderate infection. As expected, we found a negative correlation between ATP levels and RSV Ct values (RT-PCR) in NPA samples. The heightened expression of the P2X7R on cTfh cells, coupled with the decrease levels of its soluble form in plasma and NPA of children with severe RSV, may suggest an up-regulation of this pathway leading to prolonged stimulation and impairment of cTfh cells. P2X7R can be released into the blood through proteolytic cleavage (53) and can also be associated with microvesicles (36, 54). However, few studies have investigated whether the levels of sP2X7R in plasma correlate with disease severity. Garcia-Villalba et al. (37) demonstrated that plasma levels of sP2X7R in COVID-19 patients are correlated with severe disease. More recently, Vultaggio-Poma et al. (55) reported that elevated sP2X7R levels in the early phases of COVID-19 predict adverse clinical outcomes. They measured plasma levels of sP2X7R in six subgroups of COVID-19 patients, including those with symptoms at admission, those requiring transfer to the Pneumology Division, those requiring ICU admission, those requiring mechanical ventilation, patients who died during hospitalization, and patients who died after hospital discharge. Elevated sP2X7R levels were found in most of these subgroups, except in patients admitted to the ICU or those who died after hospital discharge. Conversely, Di Vicenzo et al. reported an inverse relationship between serum concentrations of sP2X7R and levels of C-reactive protein, TNF-α, and IL-6 in obese patients (56). Our results showed an increased expression of P2X7R on the surface of cTfh cells from children with severe RSV, a phenotype not associated with increased plasma levels of sP2X7R. Indeed, high levels of sP2X7R in both plasma and NPA samples were found in patients with moderate disease but not in those with severe disease. A deficiency in shedding mechanisms or in the transfer of P2X7R could potentially explain the lower sP2X7R levels and the exacerbated inflammation observed during severe RSV disease. Given the limited information available, the pathophysiological significance of P2X7R shedding and the mechanisms underlying this observation remain to be elucidated.

Remarkably, upon stimulation with anti-CD2/CD3/CD28-coated beads, PBMCs from infected children released higher levels of ATP compared to healthy donors, with the maximum release observed in PBMCs from children with severe disease. The involvement of the ATP/P2X7R pathway in the modulation of Tfh compartment is further supported by the increased frequency of P2X7R+ cTfh cells in infected children. Additionally, the P2X7R agonist BzATP demonstrated the ability to induce cTfh apoptosis and reduce the expression of the proliferation marker Ki67 and IL-21 in cTfh from infected children that were partially restored by the KN-62 antagonist. The positive correlation between disease severity and ATP levels in plasma and NPA samples, coupled with the negative correlation with cTfh frequency, suggests the potential involvement of the ATP/P2X7R pathway in both modulating the antibody response and promoting disease severity in children with RSV.

There are a number of limitations in our study. This study was conducted in a specific region of our country, so we cannot assume that our patient cohorts adequately represent the broader population of the country. It is important to emphasize that our cohort did not include any children under mild RSV infection. Additionally, the small blood sample size collected from patients limited the ability to perform multiple studies on the same specimen. We were also unable to characterize the mechanism through which ATP might modulate cTfh function in children infected with RSV. Finally, further studies should address the pathological significance of sP2X7R levels.

These findings might be relevant not only for a comprehensive understanding of the B cell response during RSV infection but also for gaining insights into the production of neutralizing antibodies in various infectious disease linked to tissue damage and the extracellular release of ATP.

## Data availability statement

The original contributions presented in the study are included in the article/**Supplementary Material**. Further inquiries can be directed to the corresponding author.

## Ethics statement

The studies involving humans were approved by the ethics committee at the “Hospital de Pediatría Pedro de Elizalde”, Buenos Aires, Argentina. The studies were conducted in accordance with the local legislation and institutional requirements. Written informed consent for participation in this study was provided by the participants’ legal guardians/next of kin.

## Author contributions

CR: Writing – original draft, Methodology, Investigation, Formal analysis, Conceptualization. SR: Writing – original draft, Supervision, Resources. SA: Writing – original draft, Supervision, Data curation. MJB: Writing – review & editing, Supervision, Resources, Data curation. NC: Writing – review & editing, Data curation. MS: Writing – review & editing, Validation, Resources. HC: Writing – review & editing. LL: Writing – review & editing, Methodology. IM: Writing – review & editing. VS: Writing – review & editing, Methodology. AO: Writing – review & editing, Methodology, Formal analysis. IS: Writing – review & editing, Methodology, Formal analysis. NS: Writing – review & editing. GA: Writing – review & editing, Methodology. GR: Writing – review & editing, Methodology. NM: Writing – review & editing, Methodology, Data curation. LP: Writing – review & editing. MH: Writing – review & editing, Methodology. MB: Writing – review & editing, Methodology. MP: Writing – review & editing, Methodology. FF: Writing – review & editing. JG: Writing – review & editing, Supervision, Investigation. LA: Writing – review & editing, Writing – original draft, Supervision, Resources, Methodology, Investigation, Funding acquisition, Formal analysis, Conceptualization.

## References

[1] Shi T McAllister DA O’Brien KL Simoes EAF Madhi SA Gessner BD. Global, regional, and national disease burden estimates of acute lower respiratory infections due to respiratory syncytial virus in young children in 2015: A systematic review and modelling study. Lancet. (2017) 390:946–58. doi: 10.1016/S0140-6736(17)30938-8 PMC5592248 28689664

[2] Srikantiah P Vora P Klugman KP. Assessing the full burden of respiratory syncytial virus in young infants in low- and middle-income countries: the importance of community mortality studies. Clin Infect Dis. (2021) 73(suppl_3):S177–9. doi: 10.1093/cid/ciab486 PMC8411251 34472571

[3] Buchwald AG Graham BS Traore A Haidara FC Chen M Morabito K. Respiratory syncytial virus (Rsv) neutralizing antibodies at birth predict protection from rsv illness in infants in the first 3 months of life. Clin Infect Dis. (2021) 11):e4421–e7. doi: 10.1093/cid/ciaa648 PMC8662775 32463443

[4] Capella C Chaiwatpongsakorn S Gorrell E Risch ZA Ye F Mertz SE. and disease severity in infants and young children with acute respiratory syncytial virus infection. J Infect Dis. (2017) 216:1398–406. doi: 10.1093/infdis/jix489 PMC5853469 29029312

[5] Chu HY Steinhoff MC Magaret A Zaman K Roy E Langdon G. Respiratory syncytial virus transplacental antibody transfer and kinetics in mother-infant pairs in Bangladesh. J Infect Dis. (2014) 210:1582–9. doi: 10.1093/infdis/jiu316 PMC4334795 24903663

[6] Mazur NI Terstappen J Baral R Bardaji A Beutels P Buchholz UJ. Respiratory syncytial virus prevention within reach: the vaccine and monoclonal antibody landscape. Lancet Infect Dis. (2023) 23:e2–e21. doi: 10.1016/S1473-3099(22)00291-2 35952703 PMC9896921

[7] Kampmann B Madhi SA Munjal I Simoes EAF Pahud BA Llapur C. Bivalent prefusion F vaccine in pregnancy to prevent rsv illness in infants. N Engl J Med. (2023) 388:1451–64. doi: 10.1056/NEJMoa2216480 37018474

[8] Harris E. Fda approves maternal rsv vaccine. Jama. (2023) 330:1029. doi: 10.1001/jama.2023.16106 37647088

[9] Crotty S. T follicular helper cell biology: A decade of discovery and diseases. Immunity. (2019) 50:1132–48. doi: 10.1016/j.immuni.2019.04.011 PMC6532429 31117010

[10] Lartey S Zhou F Brokstad KA Mohn KG-I Slettevoll SA Pathirana RD. Live-attenuated influenza vaccine induces tonsillar follicular T helper cell responses that correlate with antibody induction. J Infect Dis. (2019) 221:21–32. doi: 10.1093/infdis/jiz321 PMC6910880 31250024

[11] Morita R Schmitt N Bentebibel SE Ranganathan R Bourdery L Zurawski G. Human blood cxcr5(+)Cd4(+) T cells are counterparts of T follicular cells and contain specific subsets that differentially support antibody secretion. Immunity. (2011) 34:108–21. doi: 10.1016/j.immuni.2010.12.012 PMC3046815 21215658

[12] MacLeod MK David A McKee AS Crawford F Kappler JW Marrack P. Memory cd4 T cells that express cxcr5 provide accelerated help to B cells. J Immunol. (2011) 186:2889–96. doi: 10.4049/jimmunol.1002955 PMC3069687 21270407

[13] Brenna E Davydov AN Ladell K McLaren JE Bonaiuti P Metsger M. Cd4(+) T follicular helper cells in human tonsils and blood are clonally convergent but divergent from non-tfh cd4(+) cells. Cell Rep. (2020) 30:137–52 e5. doi: 10.1016/j.celrep.2019.12.016 31914381 PMC7029615

[14] Song W Craft J. T follicular helper cell heterogeneity: time, space, and function. Immunol Rev. (2019) 288:85–96. doi: 10.1111/imr.12740 30874350 PMC6422039

[15] Locci M Havenar-Daughton C Landais E Wu J Kroenke MA Arlehamn CL. Human circulating pd-1+Cxcr3-cxcr5+ Memory tfh cells are highly functional and correlate with broadly neutralizing hiv antibody responses. Immunity. (2013) 39:758–69. doi: 10.1016/j.immuni.2013.08.031 PMC3996844 24035365

[16] Banga R Procopio FA Noto A Pollakis G Cavassini M Ohmiti K. Pd-1(+) and follicular helper T cells are responsible for persistent hiv-1 transcription in treated aviremic individuals. Nat Med. (2016) 22:754–61. doi: 10.1038/nm.4113 27239760

[17] Moody MA Pedroza-Pacheco I Vandergrift NA Chui C Lloyd KE Parks R. Immune perturbations in hiv-1-infected individuals who make broadly neutralizing antibodies. Sci Immunol. (2016) 1:aag0851. doi: 10.1126/sciimmunol.aag0851 28783677 PMC5589960

[18] Pallikkuth S de Armas LR Rinaldi S George VK Pan L Arheart KL. Dysfunctional peripheral T follicular helper cells dominate in people with impaired influenza vaccine responses: results from the florah study. PloS Biol. (2019) 17:e3000257. doi: 10.1371/journal.pbio.3000257 31100059 PMC6542545

[19] Haltaufderhyde K Srikiatkhachorn A Green S Macareo L Park S Kalayanarooj S. Activation of peripheral T follicular helper cells during acute dengue virus infection. J Infect Dis. (2018) 218:1675–85. doi: 10.1093/infdis/jiy360 PMC6927865 29917084

[20] Sananez I Raiden SC Algieri SC Uranga M Grisolía NA Filippo D. A poor and delayed anti-sars-cov2 igg response is associated to severe covid-19 in children. EBioMedicine. (2021) 72:103615. doi: 10.1016/j.ebiom.2021.103615 34649078 PMC8502533

[21] Wilhelm K Ganesan J Muller T Durr C Grimm M Beilhack A. Graft-versus-host disease is enhanced by extracellular atp activating P2x7r. Nat Med. (2010) 16:1434–8. doi: 10.1038/nm.2242 21102458

[22] Eltzschig HK Sitkovsky MV Robson SC. Purinergic signaling during inflammation. N Engl J Med. (2012) 367:2322–33. doi: 10.1056/NEJMra1205750 PMC3675791 23234515

[23] Proietti M Cornacchione V Rezzonico Jost T Romagnani A Faliti CE Perruzza L. Atp-gated ionotropic P2x7 receptor controls follicular T helper cell numbers in Peyer’s patches to promote host-microbiota mutualism. Immunity. (2014) 41:789–801. doi: 10.1016/j.immuni.2014.10.010 25464855

[24] D’Addio F Vergani A Potena L Maestroni A Usuelli V Ben Nasr M. P2x7r mutation disrupts the nlrp3-mediated th program and predicts poor cardiac allograft outcomes. J Clin Invest. (2018) 128:3490–503. doi: 10.1172/JCI94524 PMC6063506 30010623

[25] Frascoli M Marcandalli J Schenk U Grassi F. Purinergic P2x7 receptor drives T cell lineage choice and shapes peripheral gammadelta cells. J Immunol. (2012) 189:174–80. doi: 10.4049/jimmunol.1101582 22649196

[26] Schenk U Frascoli M Proietti M Geffers R Traggiai E Buer J. Atp inhibits the generation and function of regulatory T cells through the activation of purinergic P2x receptors. Sci Signal. (2011) 4:ra12. doi: 10.1126/scisignal.2001270 21364186

[27] Atarashi K Nishimura J Shima T Umesaki Y Yamamoto M Onoue M. Atp drives lamina propria T(H)17 cell differentiation. Nature. (2008) 455:808–12. doi: 10.1038/nature07240 18716618

[28] Killeen ME Ferris L Kupetsky EA Falo L Jr. Mathers AR. Signaling through purinergic receptors for atp induces human cutaneous innate and adaptive th17 responses: implications in the pathogenesis of psoriasis. J Immunol. (2013) 190:4324–36. doi: 10.4049/jimmunol.1202045 PMC3622186 23479230

[29] Pandolfi JB Ferraro AA Sananez I Gancedo MC Baz P Billordo LA. Atp-induced inflammation drives tissue-resident th17 cells in metabolically unhealthy obesity. J Immunol. (2016) 196:3287–96. doi: 10.4049/jimmunol.1502506 26951799

[30] Piedra PA Hause AM Aideyan L. Respiratory syncytial virus (Rsv): neutralizing antibody, a correlate of immune protection. Methods Mol Biol. (2016) 1442:77–91. doi: 10.1007/978-1-4939-3687-8_7 27464689

[31] Boukhvalova MS Mbaye A Kovtun S Yim KC Konstantinova T Getachew T. Improving ability of rsv microneutralization assay to detect G-specific and cross-reactive neutralizing antibodies through immortalized cell line selection. Vaccine. (2018) 36:4657–62. doi: 10.1016/j.vaccine.2018.06.045 29960801

[32] Russo C Raiden S Algieri S De Carli N Davenport C Sarli M. Extracellular atp and imbalance of cd4+ T cell compartment in pediatric covid-19. Front Cell Infect Microbiol. (2022) 12:893044. doi: 10.3389/fcimb.2022.893044 35663467 PMC9157541

[33] Wauters E Van Mol P Garg AD Jansen S Van Herck Y Vanderbeke L. Discriminating mild from critical covid-19 by innate and adaptive immune single-cell profiling of bronchoalveolar lavages. Cell Res. (2021) 31:272–90. doi: 10.1038/s41422-020-00455-9 PMC8027624 33473155

[34] Faliti CE Gualtierotti R Rottoli E Gerosa M Perruzza L Romagnani A. P2x7 receptor restrains pathogenic tfh cell generation in systemic lupus erythematosus. J Exp Med. (2019) 216:317–36. doi: 10.1084/jem.20171976 PMC6363434 30655308

[35] Grassi F. The P2x7 receptor as regulator of T cell development and function. Front Immunol. (2020) 11:1179. doi: 10.3389/fimmu.2020.01179 32587592 PMC7297980

[36] Giuliani AL Berchan M Sanz JM Passaro A Pizzicotti S Vultaggio-Poma V. The P2x7 receptor is shed into circulation: correlation with C-reactive protein levels. Front Immunol. (2019) 10:793. doi: 10.3389/fimmu.2019.00793 31031771 PMC6474289

[37] Garcia-Villalba J Hurtado-Navarro L Penin-Franch A Molina-Lopez C Martinez-Alarcon L Angosto-Bazarra D. Soluble P2x7 receptor is elevated in the plasma of covid-19 patients and correlates with disease severity. Front Immunol. (2022) 13:894470. doi: 10.3389/fimmu.2022.894470 35663992 PMC9161710

[38] Shen W Ye H Zhang X Huo L Shen J Zhu L. Elevated expansion of follicular helper T cells in peripheral blood from children with acute measles infection. BMC Immunol. (2020) 21:49. doi: 10.1186/s12865-020-00379-4 32873255 PMC7466526

[39] Gassen RB Fazolo T Nascimento de Freitas D Borges TJ Lima K Antunes GL. Il-21 treatment recovers follicular helper T cells and neutralizing antibody production in respiratory syncytial virus infection. Immunol Cell Biol. (2021) 99:309–22. doi: 10.1111/imcb.12418 33068449

[40] Spaan M Kreefft K de Graav GN Brouwer WP de Knegt RJ ten Kate FJ. Cd4+ Cxcr5+ T Cells in Chronic Hcv Infection Produce Less Il-21, yet Are Efficient at Supporting B Cell Responses. J Hepatol. (2015) 62:303–10. doi: 10.1016/j.jhep.2014.09.024 25281860

[41] Kaneko N Kuo HH Boucau J Farmer JR Allard-Chamard H Mahajan VS. Loss of bcl-6-expressing T follicular helper cells and germinal centers in covid-19. Cell. (2020) 183:143–57.e13. doi: 10.1016/j.cell.2020.08.025 32877699 PMC7437499

[42] Yu M Charles A Cagigi A Christ W Österberg B Falck-Jones S. Delayed generation of functional virus-specific circulating T follicular helper cells correlates with severe covid-19. Nat Commun. (2023) 14:2164. doi: 10.1038/s41467-023-37835-9 37061513 PMC10105364

[43] Zhang C Yan Y He H Wang L Zhang N Zhang J. Ifn-stimulated P2y13 protects mice from viral infection by suppressing the camp/epac1 signaling pathway. J Mol Cell Biol. (2019) 11:395–407. doi: 10.1093/jmcb/mjy045 30137373 PMC7107496

[44] Tsai CY Liong KH Gunalan MG Li N Lim DS Fisher DA. Type I ifns and il-18 regulate the antiviral response of primary human gammadelta T cells against dendritic cells infected with dengue virus. J Immunol. (2015) 194:3890–900. doi: 10.4049/jimmunol.1303343 25732728

[45] Correa G de ALC Fernandes-Santos C Gandini M Petitinga Paiva F Coutinho-Silva R. The purinergic receptor P2x7 role in control of dengue virus-2 infection and cytokine/chemokine production in infected human monocytes. Immunobiology. (2016) 221:794–802. doi: 10.1016/j.imbio.2016.02.003 26969484

[46] Wolk KE Lazarowski ER Traylor ZP Yu EN Jewell NA Durbin RK. Influenza a virus inhibits alveolar fluid clearance in balb/C mice. Am J Respir Crit Care Med. (2008) 178:969–76. doi: 10.1164/rccm.200803-455OC PMC2577730 18689466

[47] Pietrobon AJ Andrejew R Custodio RWA Oliveira LM Scholl JN Teixeira FME. Dysfunctional purinergic signaling correlates with disease severity in covid-19 patients. Front Immunol. (2022) 13:1012027. doi: 10.3389/fimmu.2022.1012027 36248842 PMC9562777

[48] Seror C Melki MT Subra F Raza SQ Bras M Saidi H. Extracellular atp acts on P2y2 purinergic receptors to facilitate hiv-1 infection. J Exp Med. (2011) 208:1823–34. doi: 10.1084/jem.20101805 PMC3171090 21859844

[49] Zhang L Peeples ME Boucher RC Collins PL Pickles RJ. Respiratory syncytial virus infection of human airway epithelial cells is polarized, specific to ciliated cells, and without obvious cytopathology. J Virol. (2002) 76:5654–66. doi: 10.1128/jvi.76.11.5654-5666.2002 PMC137037 11991994

[50] Tristram DA Hicks W Jr. Hard R. Respiratory syncytial virus and human bronchial epithelium. Arch Otolaryngol Head Neck Surg. (1998) 124:777–83. doi: 10.1001/archotol.124.7.777 9677113

[51] Johnson JE Gonzales RA Olson SJ Wright PF Graham BS. The histopathology of fatal untreated human respiratory syncytial virus infection. Mod Pathol. (2007) 20:108–19. doi: 10.1038/modpathol.3800725 17143259

[52] Davis IC Sullender WM Hickman-Davis JM Lindsey JR Matalon S. Nucleotide-mediated inhibition of alveolar fluid clearance in balb/C mice after respiratory syncytial virus infection. Am J Physiol Lung Cell Mol Physiol. (2004) 286:L112–20. doi: 10.1152/ajplung.00218.2003 12948936

[53] Young CNJ Chira N Rog J Al-Khalidi R Benard M Galas L. Sustained activation of P2x7 induces mmp-2-evoked cleavage and functional purinoceptor inhibition. J Mol Cell Biol. (2018) 10:229–42. doi: 10.1093/jmcb/mjx030 28992079

[54] Pizzirani C Ferrari D Chiozzi P Adinolfi E Sandona D Savaglio E. Stimulation of P2 receptors causes release of il-1beta-loaded microvesicles from human dendritic cells. Blood. (2007) 109:3856–64. doi: 10.1182/blood-2005-06-031377 17192399

[55] Vultaggio-Poma V Sanz JM Amico A Violi A Ghisellini S Pizzicotti S. The shed P2x7 receptor is an index of adverse clinical outcome in covid-19 patients. Front Immunol. (2023) 14:1182454. doi: 10.3389/fimmu.2023.1182454 37215142 PMC10196164

[56] Di Vincenzo A Granzotto M Graziani A Crescenzi M Foletto M Prevedello L. Soluble P2x7 Receptor Plasma Levels in Obese Subjects before and after Weight Loss Via Bariatric Surgery. Int J Mol Sci. (2023) 24(23):16741. doi: 10.3390/ijms242316741 38069064 PMC10706616

