# Supplementary material for: ATP-P2X7R pathway activation limits the Tfh cell compartment during pediatric RSV infection
Source: Front Immunol. 2024 Jul 9;15:1397098. doi: 10.3389/fimmu.2024.1397098 (PMC11263008; doi:10.3389/fimmu.2024.1397098)
Supplement: Supplementary file 1 [file DataSheet_1.docx]

Supplementary Material

**ATP-P2X7R pathway activation limits the Tfh cell compartment during pediatric RSV infection**

Constanza Russo, Silvina Raiden, Silvia Algieri, Maria Jose Bruera, Norberto De Carli, Mariam Sarli, Hector Cairoli, Leonardo De Lillo, Ivanna Morales, Vanesa Seery, Adrián Otero, Inés Sananez, Nancy Simaz, Gisela Alfiero, Gabriela Rubino, Nestor Moya, Luisa Aedo Portela, Mauro Herrero, Marina Blanco, Misael Salcedo Pereira, Fernando Ferrero, Jorge Geffner, Lourdes Arruvito*

*** Correspondence:** Corresponding Author: [arruvitol@gmail.com](mailto:arruvitol@gmail.com); larruvito@fmed.uba.ar


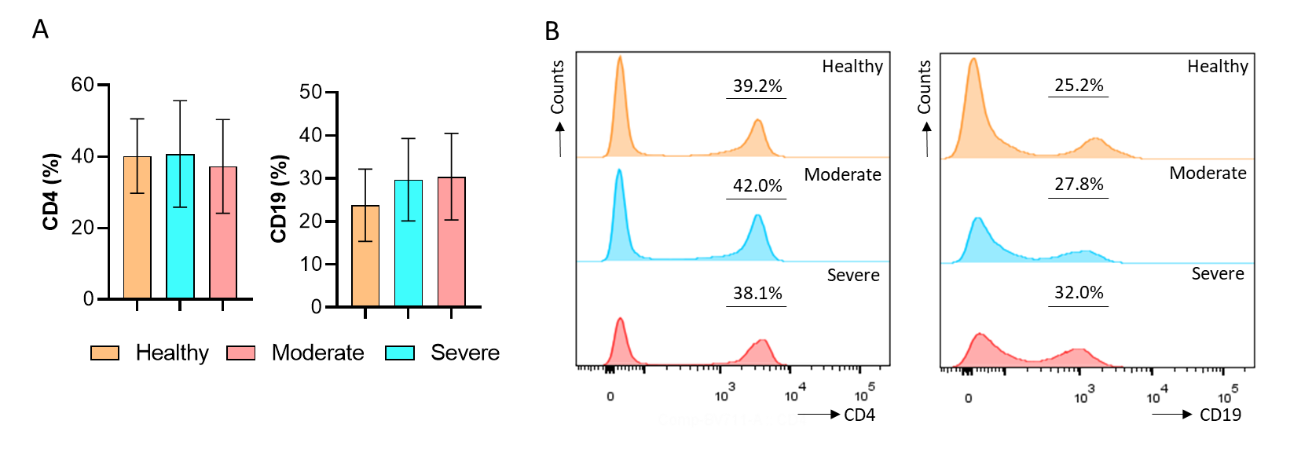


**Supplementary Figure 1.** **Frequency of T and B cells.** (A) Frequency of CD4+ T cells and CD19+ B cells in peripheral blood mononuclear cells from healthy children (n=23), children with moderate (30) and severe RSV infection (n=21) by flow cytometry. Data were acquired using a Northern Lights (Cytek) flow cytometer and analyzed with FlowJo 10.6.2. P values were determined by Kruskall-Wallis test followed by Dunn’s multiple comparison test. (B) Representative histogram of a donor from each cohort is shown.

|  | **B Coefficient** | **Standard Error** | **z-value** | **p-value** |
| --- | --- | --- | --- | --- |
| **Intercept** | 0.922 | 1.274 | 0.724 | 0.469 |
| **Age (months)** | 0.017 | 0.086 | 0.208 | 0.835 |
| **Gender (female/male)** | -0.313 | 0.975 | -0.322 | 0.072 |
| **cTfh cells (%)** | -0.294 | 0.136 | -2.159 | **<0.05** |
| **Plasma ATP levels (µM)** | 0.877 | 0.327 | 2.676 | **<0.01** |
| **Plasma sP2X7R levels (pg/mL)** | -0.003 | 0.002 | -1.627 | 0.103 |

**Supplementary Table 1. Logistic regression model to analyze disease severity**

**in children with RSV infection**

Logistic regression model to analyze disease severity in children with RSV infection using different predictor variables. Data were coded as follows. The dependent variable (disease severity), 1=severe and 0=moderate. The independent variables included gender (1=female and 0=male), age, percentage of cTfh cells, plasma ATP levels, and plasma soluble P2X7R levels (all as numeric variables). Estimated coefficients (B), their standard errors, z-values, and p-values are shown.
